# Move on Bikes Program: A Community-Based Physical Activity Strategy in Mexico City

**DOI:** 10.3390/ijerph16101685

**Published:** 2019-05-14

**Authors:** Catalina Medina, Martin Romero-Martinez, Sergio Bautista-Arredondo, Simón Barquera, Ian Janssen

**Affiliations:** 1 Department of Physical Activity and Healthy Lifestyles, Center for Nutrition and Health Research, National Institute of Public Health, Mexico City 14080, Mexico; catalina.medina@insp.mx; 2 Unidad de Posgrado, Universidad Nacional Autónoma de México, Mexico City 04510, Mexico; 3 Center for Research in Evaluation and Surveys, National Institute of Public Health, Mexico City 14080, Mexico; martin.romero@insp.mx; 4 Division of Health Economics, National Institute of Public Health, Cuernavaca 62100, Mexico; sbautista@insp.mx; 5 Center for Nutrition and Health Research, National Institute of Public Health, Cuernavaca 62100, Mexico; sbarquera@insp.mx; 6 Canada Research Chair in Physical Activity and Obesity, School of Kinesiology and Health Studies, Queen’s University, Kingston, ON K7L3N6, Canada

**Keywords:** physical activity, ciclovias, open streets, community interventions, Mexico

## Abstract

Open streets programs are free and multisectoral programs in which streets are temporally closed allowing access to walkers, runners, rollerbladers, and cyclists. The Move on Bikes program (by its name in Spanish Muévete en Bici) (MEB) consists of 55 km of interconnected streets in middle-high income areas of Mexico City. There is scarce evidence on the evaluation of this program in Mexico. The purposes of this study were to estimate the participation, physical activity levels among the MEB participants, and the association of the frequency of participation with sociodemographic, physical, and program characteristics. Methods: From October 2017 to July 2018, six hundred seventy-nine MEB participants were surveyed using a questionnaire that contains sociodemographic, physical, and program characteristics. A wide-angle video camera was used to estimate the average speed of each activity per event per participant. Based on the information collected by the program authorities and survey interviews, we estimated the number of participants per event. Results: On a typical MEB program day, 21,812 people participated. MEB program users accumulated an average of 221 min of moderate-to-vigorous physical activity (MVPA) per typical Sunday and 88.4% accumulated at least 150 min of MVPA. In total, 29.6% of users attended the program every Sunday. Those who were more likely to attend the program frequently included: men, those aged 41 to 64 years old, users classified as very and sufficiently active, those that used active transportation to travel to the program, and participants that came alone. Conclusions: This study provides evidence that the MEB program adds an extra 71 min/week of MVPA to more than 20,000 users.

## 1. Background

Chronic diseases are the leading cause of death worldwide [1]. They are largely attributable to modifiable risk factors, including physical inactivity [2,3]. In 2010, the World Health Organization (WHO) stipulated that adults should accumulate at least 150 min per week of moderate-to-vigorous physical activity (MVPA) in bouts of at least 10 min [4]. The proportion of Mexican adults who do not meet this recommendation increased by ~40% between 2006 and 2012 [5] and has remained stable since that time [6]. The high prevalence of physical inactivity will not change unless effective community-based physical activity programs and strategies are implemented.

Open street programs, such as the Ciclovia program [7], are a common community-based physical activity strategy. The Ciclovia program [7] is a multisectoral, free, mass participation program in which streets are temporarily closed to motorized transportation, allowing access to walkers, runners, rollerbladers, and cyclists [8]. There are more than 50 open streets programs being run in 67 cities across North America [9]. These programs are cost beneficial [10], improve social capital and security [9], reduce air pollution [11], are associated with an improved quality of life [12], reduced mortality risk [11], and appear to increase physical activity levels [9]. One of the limitations of previous evaluations of Ciclovia programs has been the lack of precision in the estimation of the number of participants [10].

Since 2007, the Ministry of Environment in partnership with 20 private and public organizations have implemented the Move on Bikes program (by its name in Spanish Muévete en Bici) (MEB) in Mexico City. The goal of the MEB is to reduce reliance on motorized vehicles while increasing physical activity levels. The MEB route consists of 55 km of interconnected streets in middle-high income areas, which are closed from 8:00 am to 2:00 pm on the first three Sundays of each month. Several free services are provided along the MEB route including first aid, hydration stations, washrooms, bicycle rentals, bicycle mechanics, and cycling classes [13]. The route connects with parks and other recreational activities [13].

The Ministry of Environment has made crude estimates of the number of MEB participants and the influence that the program has had on the use of motorized vehicles and bicycles [14]. However, there is a lack of information about the impact of the MEB program on physical activity levels and the factors that influence participation. The main objectives of this study were to estimate the participation, physical activity levels among MEB participants, and the association of the frequency of participation with sociodemographic, physical and program participation characteristics.

## 2. Methods

Data were collected from the start (8:00 am) to end (2:00 pm) of the MEB program each time it was offered (i.e., the first three Sundays of each month) from October 2017 to July 2018.

### 2.1. Estimate of the Number of Participants in the MEB Program

The program authorities arranged for the collection of information on the number of program participants at 16 observation points along the MEB route from the beginning (8:00 am) to the end (2:00 pm) of the program. The 16 observation points were spaced ~2.5 km apart. This spacing was selected to avoid over counting (e.g., counting the same individual on multiple occasions during each measurement window). The 16 observation points were as follows: Reforma Avenue at intersections with Julio Verne, Mariano Escobedo, Insurgentes, and Juárez (points 1 to 4); the Simón Bolivar traffic circle (point 5); Calzada de Guadalupe at intersections with Acero and Excelsior (points 6 to 7); Tlaxcoaque square (point 8); intersection of Durango and Veracruz (points 9); intersection of Mazatlan and Alfonso Reyes (point 10); intersections of Division del Norte with Viaducto, Cuauhtemoc, and Churubusco (points 11 to 13); intersection of Eje 7 Sur Zapata and Av. Universidad (point 14); intersections of Patriotismo with Empresa and Puente de la Morena (points 15 to 16). Volunteers were located at each observation point. They manually counted cyclists/rollerbladers/skateboarders, runners, and walkers for 15 min each hour, from the beginning to the end of the program. The official number of participants was estimated by summing the total number of cyclists/rollerbladers/skateboarders, runners, and walkers from all observation points for each of the 15 min measurement periods multiplied by four [14]. This estimate was corrected using information collected by the program authorities from January 2018 to July 2018. Based on this information, we identified that the location along the route with the highest number of participants was Reforma Avenue and Insurgentes (point 3). We assumed that most of the people who attended the program reached Reforma Avenue. In order to estimate the total number of participants who reached this point, at 11 out of 16 points (points 6 to 16 showed above), we asked 220 participants to answer the following question: “Are you going to reach Reforma Avenue during your journey?” In total, 76.3% of participants reported they would reach this point during their journey. We assumed that people who reported reaching Reforma Avenue would be counted at this observation point. Based on this assumption, we estimated the total number of participants (N) correcting the official estimate of the number of participants reported by the authorities as follows:

N = ((100 / (% people that reach Reforma Avenue) × total number of participants counted in Reforma Avenue)

Where:

N = total number of participants
% People that reach Reforma Avenue = 76.3%, and
Total number of participants counted in Reforma Avenue per day (average from January to July 2018) = 16,643 participants

### 2.2. Questionnaire of MEP Program Participants

Between October 2017 and March 2018 trained research assistants approached 871 MEB participants, at random, and asked them to complete a questionnaire; 150 (17.2%) declined and 42 (4.8%) had incomplete data, leaving a final sample of 679. Interviewers were primarily placed on Reforma Avenue, the most used street along the route, at a point where MEB participants frequently slow down or stop. The questionnaire took approximately 10 min to complete and was done using pen-and-paper or Survey Monkey on a cellphone. The questionnaire was originally developed by the Ministry of Environment to gather sociodemographic information on program participants [14]. For this study, we added some additional items to the questionnaire to assess the habitual physical activity levels of the participants and their physical activity when participants were not attending the program. The questionnaire was pilot-tested five times before implementation.

On the questionnaire participants reported their birthdate, last grade of school completed, zip code, marital status (single, married, widowed, divorced, or separated), weight, and height. Zip code was used to estimate the income of participants based on the National Institute of Statistics, Geography and Informatics database (by its acronym in Spanish: INEGI) [15,16]. Body mass index was calculated as kg/m^2^ and converted into underweight (<18.5 kg/m^2^), normal weight (18.5–24.9 kg/m^2^), overweight (25.0–29.9 kg/m^2^), and obese (≥30.0 kg/m^2^) categories [17].

The participants’ habitual physical activity was determined using the Global Physical Activity Questionnaire (GPAQ). This questionnaire assessed the time and frequency of physical activity performed during leisure time, work, and active transportation (walking or cycling) in a typical week. The GPAQ has been validated internationally [18] and adapted to Spanish [19]. The GPAQ data were cleaned in accordance with GPAQ protocol [19]. Total minutes per week spent in MVPA were determined. Participants were classified into three physical activity categories based on WHO physical activity recommendations [4]: (1) “physically inactive” if they participated in <150 min/week of moderate intensity, or <75 min/week of vigorous intensity, or an equivalent combination of the two intensities; (2) “sufficiently active” if they accumulated 150–299 min/week of moderate intensity, or 75–149 min/week of vigorous intensity, or an equivalent combination; or (3) “very active” if they participated in ≥300 min/week of moderate intensity, or ≥150 min/week of vigorous intensity, or an equivalent combination [4].

Additional questionnaire items (i.e., “What is the main activity you perform during the program?” and “How many hours do you usually spend in the program?”) were used to measure the type of physical activity (cycle, walk, jog/run, rollerblade) and the typical duration spent in the program on the day the questionnaire was completed. The intensity was obtained by estimating the average speed of participants from data collected using a wide-angle camera. The camera was placed on a pole approximately 1.50 m off the ground in the middle of the street. The street contained a mark on the route with the number of meters to the camera. Fifty participants per activity were randomly selected. We estimated the time it took each participant to cycle, walk, rollerblade, and run from the mark on the route to the camera. Average speed was calculated by the following formula: speed = distance/time. The average speed was 13.8 km/h for cycling, 4.7 km/h for walking, 9 km/h for rollerblading, and 9.8 km/h for running. The activities were classified as moderate (walking, rollerblading, and cycling) or vigorous (running) based on the intensity for these travel speeds noted in the compendium of activities [20]. Using this information, we classified participants as “physically inactive”, “sufficiently active”, or “very active” based on their program participation alone, using the previously mentioned WHO physical activity recommendations criteria [4].

We also asked questions to assess the frequency at which they participate in the program (every Sunday including the participation within the MEB (i.e., the first three Sundays of each month) and the Ciclotón program (similar program than the MEB but carried out the last Sunday of each month), twice per month, once per month, do not attend regularly, first time), their mode of travel to get to the program on the day the questionnaire was completed (car, public transportation, or active transportation (bicycle, walking, rollerblading, or jogging/running)), if they attended the program alone or were accompanied by another person on the day they completed the questionnaire (i.e., family member, co-workers, partner, neighbor, classmate, friend), and the type of activities they normally do on the Sundays where they do not attend the program (sedentary activities, active activities, or very active activities). To estimate the distance they travelled while participating in the program on the day the questionnaire was completed, participants were asked: “Where did you start your journey?”, “Where did you finish your journey?”, and “Which is the farthest point you reached during your journey?”. A route was drawn on Google MyMaps based on the connection of the three journey points (beginning–farthest–end point) and the total kilometers of each one of the journey points were added to obtain the total distance traveled.

All participants provided their written informed consent prior to participating. The National Public Health Institute Ethics Review Board of Mexico approved the study (No. 1703).

### 2.3. Statistical Analysis

Statistical analyses were performed using SPSS software version 25 (IBM SPSS statistics, IBM Corporation, Somers, NY). The level of significance was set at *p* < 0.05. Simple descriptive statistics (means, standard deviation, medians, interquartile ranges, percentages, 95% confidence intervals) were used to make inferences. Kolmogorov–Smirnov test was used to examine normality. Variables that were not normally distributed were logarithmically transformed prior to subsequent analyses. Percentages and confidence intervals were used to describe the sample based on sociodemographic, physical, and program characteristics. Logarithmic transformed mean differences between MEB minutes per week according to sociodemographic, physical, and MEB characteristics were examined using independent Student’s *t*-test and ANOVA. The *p* value used to denote statistical significance was adjusted for multiple comparisons using Bonferrioni’s method. Univariate ordinal regression analysis was used to examine the association between frequency of participation and the characteristics of the users. Multivariate ordinal regression analysis was adjusted by sex, age, educational level, habitual physical activity, and body mass index. The model was tested for assumptions before further analysis.

## 3. Results

### 3.1. Participation Numbers

**Figure 1:**
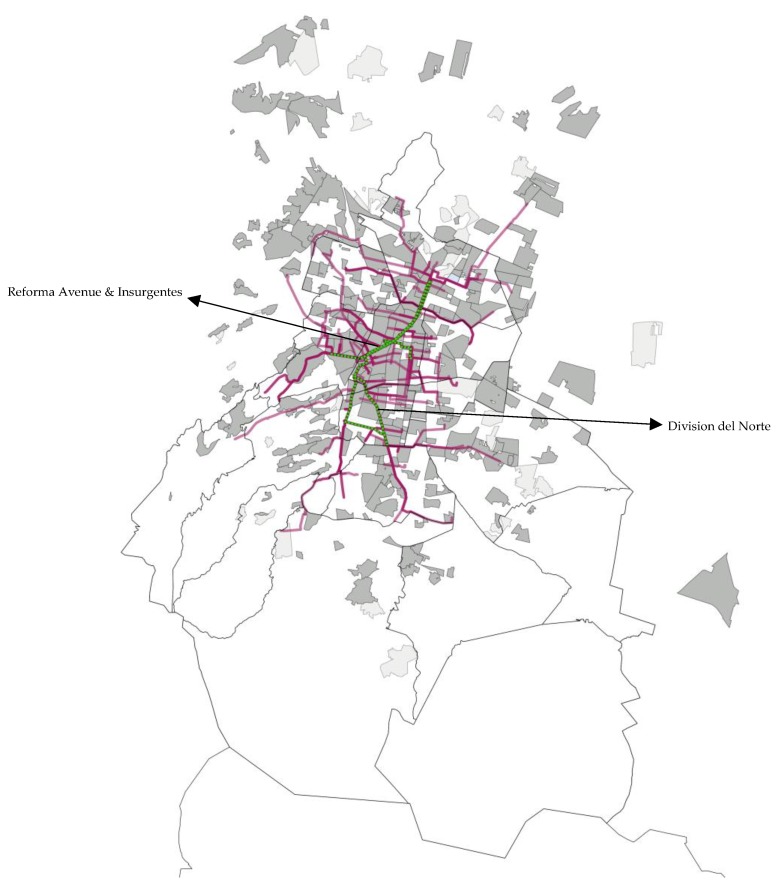
Map with the distribution of the participants and routes. México City, 2017–2018. Green line—program route; purple line—participants’ route; shadows—areas where participants live (dark shadows—people from high-income areas, light shadows—people from middle-income areas).

We estimated that 21,812 (95% CI: 20,111 to 23,491) people attended the program on the average MEB program day. Reforma Avenue, the most important and emblematic street in Mexico City, was used by more than 70% of participants at some point while participating in the program. The second most frequently used route section was Division del Norte, a route that connects the south and the north parts of Mexico City (Figure 1).

### 3.2. Characteristics of Program Participants

Sociodemographic, physical, and physical activity characteristics of the 679 MEB participants who completed the questionnaire are in Table 1. We estimated that 51.3% were women, 64.1% were between 18 to 40 years of age, 62.8% were single, 61.2% had a Master’s degree or higher, and 13% reported having obesity. Based on the reported zip code, 84.4% of MEB participants lived in Mexico City and 13.8% lived in the State of Mexico (Figure 1). The majority of participants lived in middle-income (7.6%) or high-income (91.8%) neighborhoods. During the week in which they were interviewed, 14.1% of program participants were physically inactive, 15.6% were sufficiently active, and 70.3% were very active based on WHO guidelines. Approximately 61.7% of participants accumulated enough physical activity during the program alone to meet the criteria for sufficiently active and 26.7% accumulate enough physical activity during the program alone to meet the criteria for very active. Finally, 54.8% reported that they would be engaged in sedentary or light intensity activities if they were not at the MEB program.

On average, participants accumulated 221 min of MVPA at the program on the days they participated. Information on what participants did when they attended the program is in Table 1. The majority (88.5%) reported that they cycled during the program, and approximately 57% of the cyclists did not wear a helmet. Almost 60% of participants travelled at least 10 km while participating in the program. Almost 70% came to the program with at least one other person and 39.6% indicated that they participated in the program at least twice per month.

### 3.3. Correlates of Duration and Frequency of Program Participation

As shown in Table 2, men spent more time participating in the MEB than women did (*p* < 0.05), 41–64 year olds spent more time in the program than those aged 18–40 years (*p* < 0.05), and those with a Master’s degree or higher spent less time in the program than those with a high school or Bachelor’s degree (*p* < 0.001). Those who rode a bicycle reported spending significantly more time at the MEB program compared to those who performed other activities (*p* < 0.01). People that used active transportation to travel to the program spent more time at the MEB program compared to those who travelled to the program by car or public transportation (240 vs. 180, *p* < 0.05). Finally, those that came alone and every Sunday reported a higher number of minutes in the MEB compared to those that came with someone and less frequent users (*p* < 0.05).

Table 3 shows the association between sociodemographic, physical, and program characteristics and the frequency of participation. Based on the multivariate model, men, those within the 41–64 year old group, those classified as sufficiently active or very active, those that used active transportation to travel to the program, and those that came alone were more likely to attended the program more frequently compared to their counterparts. Conversely, individuals classified as overweight and who had a master’s degree or higher attended the program less frequently compared to the normal weight and less educated group, respectively.

## 4. Discussion

The aims of this study were to estimate the number of people who participate in the MEB program, the physical activity levels of MEB program participants, and the associations between the frequency of MEB program participation with sociodemographic, physical and program characteristics. On a typical Sunday, 21,812 people participated in the program. MEB participants accumulated an average of 221 min of MVPA at the program. In total, 29.6% of the participants attend the MEB program every Sunday. Approximately 88.5% of the participants used a bicycle, 62.8% were single, and 34.2% came to the program alone. Men, those aged 41 to 64 years old, those classified as sufficiently active or very active, those that used active transportation to travel to the program, and those who came alone were more likely to attend the program frequently.

On average, 21,812 participants attended the program on a typical Sunday. Estimated participation numbers in other Ciclovia programs held around the world range from 1000 to 1,000,000 people [7,21,22,23,24,25,26]. Research suggests that the Bogotá Ciclovía Recreativa is the program with the most participants [26]. The wide variation in the number of attendees among the programs could reflect that there is no standardized method for calculating the number of participants and the approaches used in different studies have varied considerably. For instance, CicloSDias in San Diego, California estimated the number of attendees using the highest 5 h count obtained from three different stations [22]. The same methodology was used in the Saint Louis open streets and the Summer Streets in New York City [23,24]. The CicLAvia in Los Angeles used a camera that counted all the people passing through in 5 min interval [21]. Finally, the Ciclovia in a rural Latino area conducted a direct observation for 15 min each hour for a total of five time points and they were not able to calculate the total number of participants [25]. Based on the estimates from these programs, the number of participants seems to be overestimated and this overestimation could reflect double counting of participants at many of the programs. There is a wide variety in the estimation of the number of participants among the programs due to the size population of the cities, and differences between programs (promotion, duration and frequency). Thus, future research of these programs should be done using validated methodologies, which would facilitate comparisons between programs.

Based on our results, MEB users spend on average 221 min on the 34-mile route (55 km) during the 6-hour duration of the program. Participants from other Ciclovia programs spent between 56 to 180 min in a 3 to 7 h event [21,22,23,24,25,27,28,29,30,31,32]. The cities of Latin American countries have some of the longest programs ranging from 20.5 to 75 miles [9,30,32]. We found that 88.4% of the MEB users accumulated enough MVPA during the event to meet the weekly physical activity recommendations proposed by the WHO. This prevalence is higher than what has been reported in other studies. For instance, 75% of Chile [30], 39% of the San Diego [22], 19.4% of Atlanta [27], 50% of Saint Louis and 17.3% of Texas-Mexico border [23] participants meet the recommendations of physical activity during the event. The higher values reported in our program could be related to the length and frequency of the route. In addition, the higher estimate in our study may reflect that the users reported the average time they expected to stay in the program but not the time they spent active. Further studies should identify ways to objectively measure physical activity levels in these programs.

In total, ~55% of the MEB users reported that they would have been engaged in sedentary or light activities if they were not at the MEB program. This means that 47% of people who meet the weekly physical activity recommendations at the event would be classified as physically inactive if they did not attend the program. A high proportion of users in other Ciclovia programs, such as those in Atlanta (38%) [27], Saint Louis (43%) [23], and rural areas in central Washington state (79%) [25], also indicated that they would have otherwise been sedentary if there were not at the program. Thus, these programs offer a place for the general public to be active and reduce sedentarism and they appear to contribute to increased physical activity levels in the communities where they are offered. Based on previous studies, those participants who meet the physical activity recommendation and participate in open streets programs were more likely to have a high mean score of health-related quality of life [12]. However, further research is needed to determine the association between participating in open streets programs and improving quality of life and other health metrics.

We found that men, middle to older aged adults, those that cycle, those that used active transportation to travel to the program, those that came alone, and those that attended every Sunday reported spending significantly higher minutes at the program compared to their counterparts. In addition, participants classified as very and sufficiently active regularly and during the program, were more likely to participate more frequently. These results are similar to those reported by other studies [5,9,22,23,24,28,33]. Although open streets programs allow people to achieve physical activity recommendations by either biking or doing other recreational activities such as Zumba, more effort is required to get physically inactive and overweight people involved. Further research should aim to identify the main reasons why physically inactive and overweight people are less likely to participate in the program and to identify strategies that can be used to increase participation rates among these individuals.

Despite the fact that the MEB program offers strategies to encourage participation amongst people from diverse income areas, such as free public transportation and bicycle rentals, the majority of participants came from middle-to-high income areas. Moreover, people from these areas spent more time at the program upon arrival. Thus, strategies to encourage greater participation amongst people from more disadvantaged areas are needed.

Strengths of this study included the collection of sociodemographic and physical characteristics of MEB participants. In addition, our study included a wide age range (i.e., 18–87 year olds). Finally, the sample size was large enough to observe differences between groups. One of the main limitations is that our sample was recruited by convenience and the results may not be representative of all attendees. In addition, MEB participants that agreed to answer the questionnaire could be more aware of their health and physical activity levels than those who chose not to participate. The percentage of women who completed the survey was 51.3%, which is considerably lower than the 70% proportion who participate in the program. Moreover, instantaneous speed, collected by the video cameras, may not represent the average speed of participants per event. The formula we used to correct the official number of participants may not estimate the exact number of users because we assumed that all participants who reported reaching Reforma Avenue were counted at that observation point.

## 5. Conclusions

More than 20,000 people participate in the MEB program on a typical Sunday. The time spent in physical activity while participating in the MEB program contributes toward 40% of the minimum weekly physical activity target recommended by the WHO. The most frequent program users were more likely to be physically active and use a bicycle to participate in the program.

## Figures and Tables

**Table 1:** Characteristics of the Move on Bikes (by its name in Spanish Muévete en Bici) (MEB) program participants and a description of what they were doing while participating in the program. México City, 2017–2018.

Variables	N = 679 % (95% CI)
Gender	
Men	48.7 (44.9, 52.6)
Women	51.3 (47.4, 55.1)
Age (years)	
18–40	64.1 (60.3, 67.7)
41–64	33.9 (30.3, 37.6)
65–87	2.0 (1.1, 3.4)
Marital status ^◊^	
Single	62.8 (59.1, 66.5)
Partner	37.2 (33.5, 40.9)
Education level	
Primary or secondary	1.2 (0.5, 2.3)
High school or bachelor’s degree	37.6 (33.9, 41.3)
Master’s degree or higher	61.2 (57.5, 64.9)
Socioeconomic status	
Low	0.6 (0.2, 1.6)
Middle	7.6 (5.6, 9.9)
High	91.8 (89.3, 93.8)
State of Mexico	
Mexico City	84.4 (81.4, 87.0)
State of Mexico	13.8 (11.3, 16.7)
Others	1.8 (0.9, 3.1)
Body mass index category (kg/m^2^)	
Normal weight	48.6 (44.8, 52.4)
Overweight	38.4 (34.7, 42.2)
Obese	13.0 (10.6, 15.8)
Habitual physical activity level	
Physically inactive	14.1 (11.6, 17.0)
Sufficiently active	15.6 (13.0, 18.6)
Very active	70.3 (66.7, 73.7)
Activities if participants do not attend the program	
Sedentary or light	54.8 (51.0, 58.6)
Active and very active	45.2 (41.4, 49.0)
Physical activity level at the program	
Physically inactive	11.6 (9.2, 14.2)
Sufficiently active	61.7 (58.0, 65.4)
Very active	26.7 (23.4, 30.2)
Activity at the program	
Cycle	88.5 (85.9, 90.8)
Rollerblade	3.2 (2.0, 4.9)
Walk	3.1 (1.9, 4.7)
Jog/Run	5.2 (3.6, 7.1)
Distance travelled during the program (km)	
<10	40.1 (36.4, 43.9)
10 or more	59.9 (56.1, 63.6)
People accompanied by at the program *	
Came alone	34.2 (30.6, 37.9)
Came with another person	65.8 (62.1, 69.4)
Frequency of participation in the program	
First time	9.3 (7.2, 11.7)
Do not participate regularly	8.5 (6.6, 10.9)
1 time per month	13 (10.5, 15.7)
2–3 times per month	39.6 (35.9, 43.4)
Every Sunday	29.6 (26.2, 33.2)

^◊^ Marital status: partner—partner or married. Single—single, widowed, divorced, separated; * Came with another person—family, co-workers, partner, neighbors, classmates, friends; physically inactive <150 min/week of moderate-vigorous physical activity, sufficiently active 150–299 min/week of moderate-vigorous physical activity, very active ≥300 min/week of moderate-vigorous physical activity.

**Table 2:** Mean and median minutes spent at the program based on sociodemographic, physical, and program characteristics. México City, 2017–2018.

Variables	Mean (SD)	Median (IQR)^∙^
Sociodemographic characteristics		
Total	221 (72)	180 (180, 240)
Gender		
Men	229 (75)	240 (180, 300) ^b^
Women	213 (69)	180 (180, 240)
Age (years)		
18–40	213 (71)	180 (180, 240) ^b^
41–64	236 (71)	240 (180, 300)
65–87	223 (86)	240 (165, 255)
Marital status ^◊^		
Single	221 (72)	180 (180, 240)
Partner	220 (74)	180 (180, 240)
Education level		
Primary or secondary	218 (31)	240 (180, 240)
High school or bachelor’s degree	234 (75)	240 (180, 300) ^c^
Master’s degree or higher	213 (71)	180 (180, 240)
Physical characteristics		
Body mass index category (kg/m^2^)		
Normal weight	220 (72)	180 (180, 240)
Overweight	221 (72)	180 (180, 240)
Obese	224 (74)	180 (180, 300)
Habitual physical activity level		
Physically inactive	209 (67)	180 (180, 240)
Sufficiently active	213 (73)	180 (180, 240)
Very active	225 (73)	210 (180, 300)
Program characteristics		
Activity at the program		
Cycle	227 (72)	240 (180, 300) ^b, c, d^
Rollerblade	172 (43)	180 (120, 180)
Walk	186 (85)	180 (120, 240)
Jog/run	177 (62)	180 (120, 180)
Transportation to the program ^†^		
Car	201 (65)	180 (180, 240)
Public transportation	209 (69)	180 (180, 240)
Active transportation	234 (74)	240 (180, 300) ^a, b^
People accompanied by at the program *		
Came alone	236 (74)	240 (180, 300) ^b^
Came with another person	213 (70)	180 (180, 240)
Frequency of participation in the program		
First time	186 (58)	180 (120, 240) ^e^
Do not participate regularly	211 (70)	180 (180, 240) ^e^
1 time per month	194 (64)	180 (180, 240) ^e^
2–3 times per month	225 (73)	240 (180, 300) ^a, c^
Every Sunday	241 (73)	240 (180, 300)

**^∙^** IQR: 25th–75th percentile; ^◊^ Marital status: partner—partner or married. Single—single, widowed, divorced, separated; ^†^ Active transportation—cycling, running, walking; * Came with another person—family, co-workers, partner, neighbors, classmates, friends; physically inactive <150 min/week of moderate-vigorous physical activity, sufficiently active 150–299 min/week of moderate-vigorous physical activity, very active ≥300 min/week of moderate-vigorous physical activity. ^a, b, c, d^ Different superscript letters indices represent statistically significant differences between characteristics (^a^ men vs. ^b^ women; ^a^ 18–40 vs. ^b^ 41–64 vs. ^c^ 65–87 years; ^a^ single vs. ^b^ partner; ^a^ primary or secondary vs. ^b^ high school or Bachelor’s degree vs. ^c^ Master’s degree or higher; ^a^ normal weight vs. ^b^ overweight vs. ^c^ obese; ^a^ physically inactive vs. ^b^ sufficiently active vs. ^c^ very active; ^a^ cycle vs. ^b^ rollerblade vs. ^c^ walk vs ^d^ jog/run; ^a^ car vs. ^b^ public transportation vs. ^c^ active transportation; ^a^ came alone vs. ^b^ came with another person; and ^a^ first time vs. ^b^ do not participate regularly vs. ^c^ 1 time per month vs. ^d^ 2–3 times per month vs. ^e^ every Sunday) (*p* < 0.05).

**Table 3:** Ordinal regression analysis for the association between the frequency of attendance and sociodemographic, physical, and program characteristics.

Variables	Univariate Model OR^∙^ (95% CI)	Multivariate Model OR^∙^ (95% CI)
Sociodemographic characteristics		
Gender		
Women	1	1
Men	**1.60 (1.21, 2.10)**	**1.40 (1.05, 1.87)**
Age (years)		
18–40	1	1
41–64	**3.01 (2.22, 4.08)**	**2.85 (2.08, 3.90)**
65–87	**3.75 (1.36, 10.36)**	2.23 (0.75, 6.66)
Marital status ^◊^		
Single	1	1
Partner	1.06 (0.80, 1.41)	0.86 (0.63, 1.17)
Education level		
Primary or secondary	1	1
High school or bachelor’s degree	**0.09 (0.01, 0.68)**	0.15 (0.02, 1.19)
Master’s degree or higher	**0.05 (0.01, 0.39)**	**0.10 (0.01, 0.77)**
Physical characteristics		
Body mass index category (kg/m^2^)		
Normal weight	1	1
Overweight	0.90 (0.67, 1.21)	**0.70 (0.51, 0.95)**
Obesity	1.02 (0.67, 1.57)	0.68 (0.43, 1.06)
Habitual physical activity prevalence		
Physically inactive	1	1
Sufficiently active	**1.87 (1.13, 3.08)**	**1.73 (1.04, 2.88)**
Very active	**2.47 (1.66, 3.68)**	**2.19 (1.45, 3.29)**
Program characteristics		
Physical activity prevalence at the program		
Physically inactive	1	1
Sufficiently active	**2.23 (1.44, 3.46)**	**1.88 (1.21, 2.93)**
Very active	**4.53 (2.77, 7.40)**	**3.59 (2.18, 5.92)**
Transportation to the program		
Car	1	1
Public transportation	0.90 (0.60, 1.36)	0.93 (0.61, 1.42)
Active transportation ^†^	**3.27 (2.28, 4.68)**	**2.45 (1.69, 3.57)**
People accompanied by at the program		
Came with another person *	1	1
Came alone	**2.25 (1.67, 3.03)**	**1.89 (1.38, 2.58)**

**^∙^** OR: odds ratio; Bold: significant differences; Multivariate model adjusted for sex, age, educational level, physical activity levels, and body mass index; ^◊^ Marital status: partner—partner or married. Single—single, widowed, divorced, separated; ^†^ Active transportation—cycling, running, walking; * Came with another person—family, co-workers, partner, neighbors, classmates, friends; physically inactive <150 min/week of moderate-vigorous physical activity, sufficiently active 150–299 min/week of moderate-vigorous physical activity, very active ≥300 min/week of moderate-vigorous physical activity.

## Data Availability

The dataset generated during the current study is not publicly available because the National Institute of Public Health and The Ministry of Mobility of Mexico City own it, but is available from the corresponding author on reasonable request.
